# Crystal structure and Hirshfeld surface analysis of (*E*)-6-(4-hy­droxy-3-meth­oxy­styr­yl)-4,5-di­hydro­pyridazin-3(2*H*)-one

**DOI:** 10.1107/S2056989019014130

**Published:** 2019-10-31

**Authors:** Said Daoui, Cemile Baydere, Fouad El Kalai, Rafik Saddik, Necmi Dege, Khalid Karrouchi, Noureddine Benchat

**Affiliations:** a Laboratory of Applied Chemistry and Environment (LCAE), Faculty of Sciences, Mohamed I University, 60000 Oujda, Morocco; b Department of Physics, Faculty of Arts and Sciences, Ondokuz Mayıs University, 55139 Samsun, Turkey; c Laboratory for Organic Synthesis, Extraction and Valorization, Faculty of Sciences, Ain Chok, University Hassan II, Casablanca, Rabat, Morocco; d Laboratory of Plant Chemistry, Organic and Bioorganic Synthesis, URAC23, Faculty of Science, BP 1014, GEOPAC Research Center, Mohammed V University, Rabat, Morocco

**Keywords:** crystal structure, hydrogen bonding, Hirshfeld surface analysis, pyridazine

## Abstract

In the title com­pound, C_13_H_14_N_2_O_3_, the dihydropyridazine ring (r.m.s. deviation = 0.166 Å) has a screw-boat conformation. The dihedral angle between its mean plane and the benzene ring is 0.77 (12)°. In the crystal, inter­molecular O—H⋯O hydrogen bonds generate *C*(5) chains and N—H⋯O hydrogen bonds produce *R*_2_^2^(8) motifs. These types of inter­actions lead to the formation of layers parallel to (12). The three-dimensional network is achieved by C—H⋯O inter­actions, including *R*_2_^4^(8) motifs. Inter­molecular inter­actions were additionally investigated using Hirshfeld surface analysis and two-dimensional fingerprint plots. The most significant contributions to the crystal packing are by H⋯H (43.3%), H⋯C/C⋯H (19.3%), H⋯O/H⋯O (22.6%), C⋯N/N⋯C (3.0%) and H⋯N/N⋯H (5.8%) contacts. C—H⋯π inter­actions and aromatic π–π stacking inter­actions are not observed.

## Abstract 2

In the title com­pound, inter­molecular C—H⋯O, O—H⋯O and N—H⋯O hydrogen bonds link the mol­ecules into a three-dimensional supra­molecular network.

## Chemical context   

For decades the chemistry of pyridazinones has been an inter­esting field. This nitro­gen heterocycle became a scaffold of choice for the development of potential drug candidates (Akhtar *et al.*, 2016 ▸; Dubey & Bhosle, 2015 ▸) because pyridazinone and its substituted derivatives are important pharmacophores possessing many different biological applications (Asif, 2014 ▸). Such com­pounds are used as anti-HIV (Livermore *et al.*, 1993 ▸), anti­microbial (Sönmez *et al.*, 2006 ▸), anti­convulsant (Partap *et al.*, 2018 ▸), anti­hypertensive (Siddiqui *et al.*, 2011 ▸), anti­depressant (Boukharsa *et al.*, 2016 ▸), analgesic (Gökçe *et al.*, 2009 ▸), anti-inflammatory (Barberot *et al.*, 2018 ▸), anti­histaminic (Tao *et al.* 2012 ▸), cardiotonic (Wang *et al.*, 2008 ▸) and herbicidal agents (Asif, 2013 ▸) or as glucan synthase inhibitors (Zhou *et al.*, 2011 ▸).
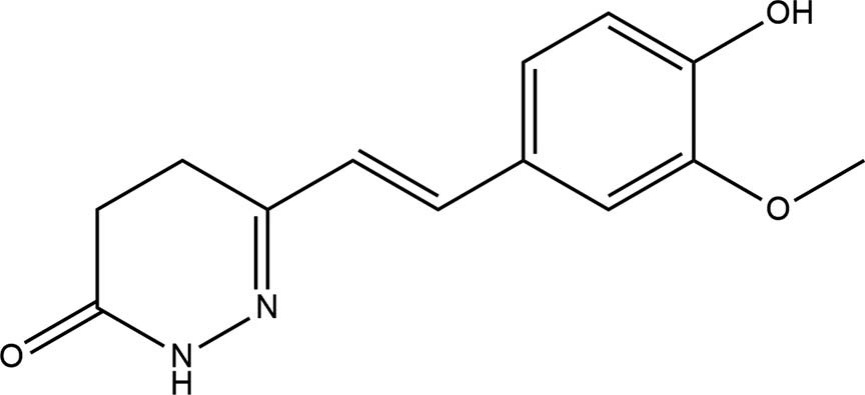


In continuation of our studies related to mol­ecular structures and Hirshfeld surface analysis of new heterocyclic derivatives (Daoui *et al.*, 2019*a* ▸,*b* ▸; El Kalai *et al.*, 2019 ▸; Karrouchi *et al.*, 2015 ▸), we report herein on the synthesis, mol­ecular and crystal structures of (*E*)-6-(4-hy­droxy-3-meth­oxy­styr­yl)-4,5-di­hydro­pyridazin-3(2*H*)-one, as well as an analysis of the Hirshfeld surfaces.

## Structural commentary   

**Figure 1:**
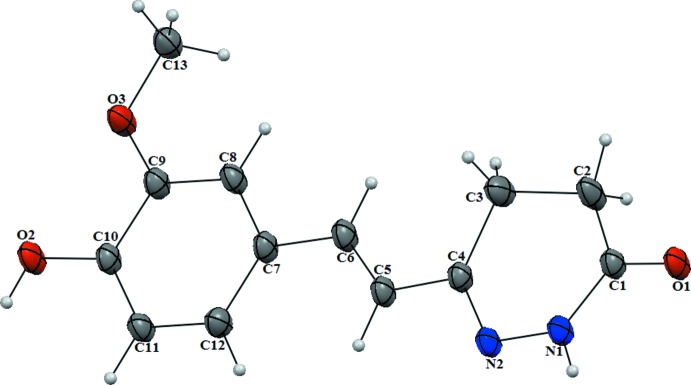
The mol­ecular structure of the title com­pound. Displacement ellipsoids are drawn at the 50% probability level.

In the title mol­ecule (Fig. 1 ▸), the configuration relative to the double bond at C5 and C6 is *E*. The dihydropyridazine ring has a screw-boat conformation, with an r.m.s. deviation of 0.166 Å for the ring atoms, with the maximum deviation from the ring being 0.178 (3) Å for the C3 atom; the C2 atom lies −0.177 (3) Å out of the plane in the opposite direction relative to the C3 atom. The dihedral angle between the dihydropyridazine ring mean plane and the benzene ring (C7–C12) is 0.77 (12)°, indicating an almost planar conformation of the molecule favouring delocalization over the C4—C5=C6—C7 bridge.

## Supra­molecular features   

**Figure 2:**
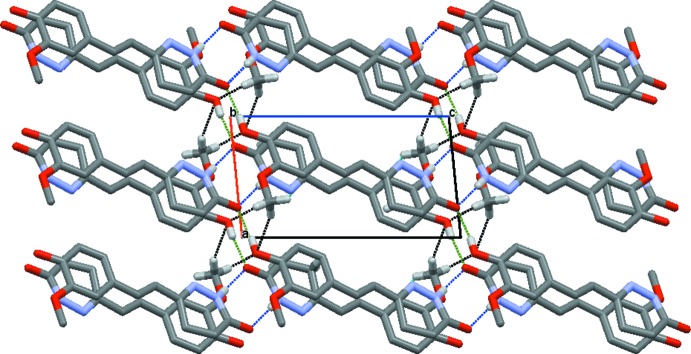
The crystal packing of the title com­pound, with N—H⋯O, O—H⋯O and C—H⋯O inter­actions shown as blue, green and black dashed lines, respectively.

In the crystal, mol­ecules are stacked in rows parallel to [100]. Notably, no significant C—H⋯π or π–π inter­actions are observed. O2—H2⋯O1^i^ hydrogen bonds between the phenolic OH group and the carbonyl O atom of a neighbouring mol­ecule generate *C*(5) chains extending parallel to [101]. Likewise, N1—H1⋯O1^ii^ hydrogen bonds between the N—H function of the di­hydro­pyridazine ring and the carbonyl O atom generate centrosymmetric dimers with an 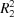
(8) motif. The two types of hydrogen bonding result in the formation of layers parallel to (12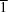). A three-dimensional supra­molecular network is eventually formed through inter­molecular C13—H13*A*⋯O2^iii^ and C13—H13*C*⋯O2^iv^ hydrogen bonds with 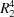
(8) motifs (Fig. 2 ▸ and Table 1 ▸).

## Database survey   

A search of the Cambridge Structural Database (CSD, Version 5.40, update November 2018; Groom *et al.*, 2016 ▸) revealed two structures containing a similar pyridazinone moiety as in the title structure but with different substituents, *viz*. 6-phenyl-4,5-di­hydro­pyridazin-3(2*H*)-one (CSD refcode TADQUL; Abourichaa *et al.*, 2003 ▸) and (*R*)-(−)-6-(4-amino­phen­yl)-5-methyl-4,5-di­hydro­pyridazin-3(2*H*)-one (ADIGOK; Zhang *et al.*, 2006 ▸). In the structure of TADQUL, the di­hydro­pyridazine ring adopts a half-chair conformation, with atoms C1, N2, N3 and C4 in a common plane, with C5 0.222 (2) Å and C6 0.262 (2) Å on opposite sides of this plane. The plane is almost coplanar with the 4-aminophenyl ring, the dihedral angle between the two planes being 1.73 (9) Å. In the crystal, hydrogen-bonded centrosymmetric dimers are observed. The O1=C1 bond length is 1.2316 (14) Å. The N3—C4, N2—N3 and N2—C1 bond lengths are 1.3464 (15), 1.3877 (14) and 1.2830 (15) Å, respectively. In the structure of ADIGOK, the asymmetric unit consists of two mol­ecules of the same enanti­omer, and the crystal packing is stabilized by inter­molecular N—H⋯O hydrogen bonds.

## Hirshfeld surface analysis   

**Figure 3:**
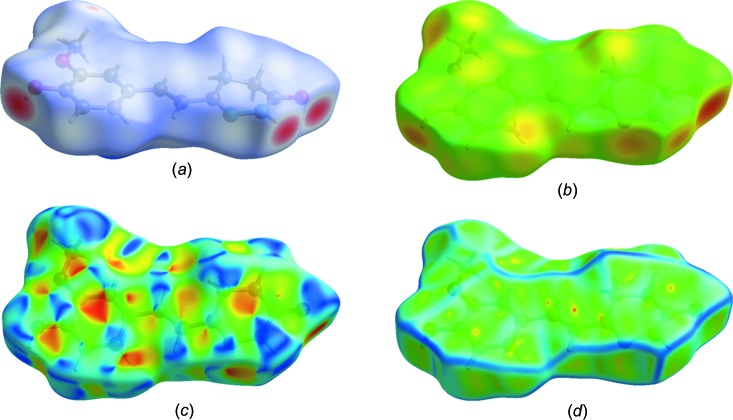
(*a*) *d*_norm_ mapped on the Hirshfeld surface for visualizing the inter­molecular inter­actions, (*b*) *d*_e_ mapped on the surface, (*c*) shape-index map of the title com­pound and (*d*) curvedness map of the title com­pound using a range from −4 to 4 Å.

**Figure 4:**
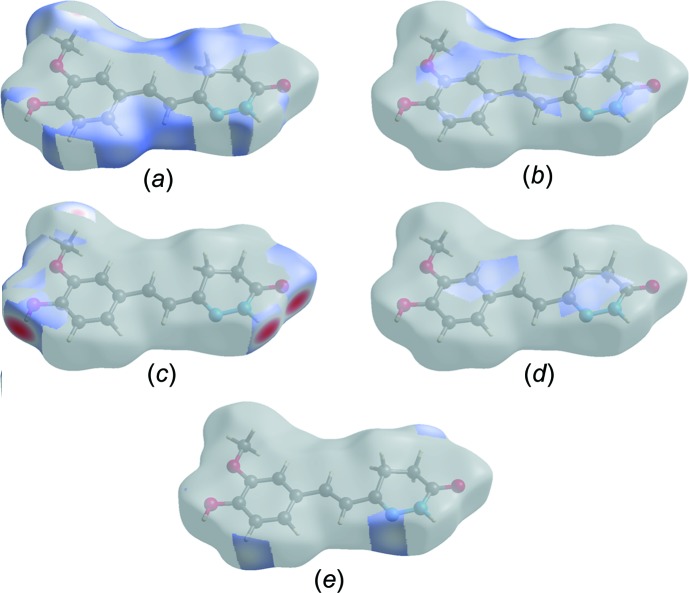
The Hirshfeld surface representations with the function *d*_norm_ plotted onto the surface for (*a*) H⋯H, (*b*) H⋯C/ C⋯H, (*c*) H⋯O/O⋯H, (*d*) C⋯N/N⋯C and (*e*) H⋯N/N⋯H inter­actions.

**Figure 5:**
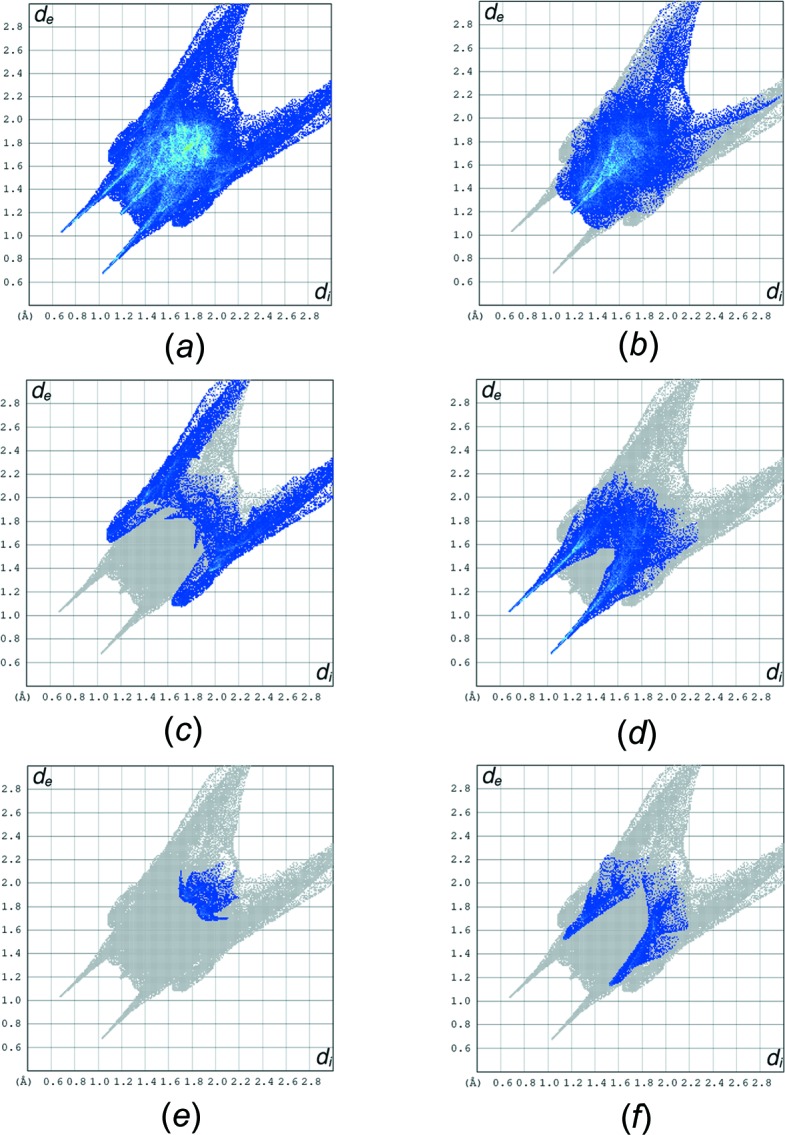
The full two-dimensional fingerprint plots for the title com­pound, showing (*a*) all inter­actions, and delineated into (*b*) H⋯H, (*c*) H⋯C/ C⋯H, (*d*) H⋯O/O⋯H, (*e*) C⋯N/N⋯C and (*f*) H⋯N/N⋯H inter­actions.

Hirshfeld surface analysis was used to qu­antify the inter­molecular inter­actions of the title com­pound, using *CrystalExplorer17.5* (Turner *et al.*, 2017 ▸). The Hirshfeld surface analysis was planned using a standard (high) surface resolution with the three-dimensional *d*_norm_ surfaces plotted over a fixed colour scale of −0.7021 (red) to 2.2382 a.u. (blue). The surfaces mapped over relevant inter­molecular contacts are illustrated in Fig. 3 ▸. The Hirshfeld surface representations with the function *d*_norm_ plotted onto the surface are shown for the H⋯H, H⋯C/C⋯H, H⋯O/O⋯H, C⋯N/N⋯C and H⋯N/N⋯H inter­actions in Figs. 4 ▸(*a*)–(*e*), respectively. The overall two-dimensional fingerprint plot and those delineated into H⋯H, H⋯C/C⋯H, H⋯O/O⋯H, C⋯N/N⋯C and H⋯N/N⋯H contacts are illustrated in Figs. 5 ▸(*a*)–(*f*), respectively. The largest inter­action is that of H⋯H, contributing 43.3% to the overall crystal packing. H⋯C/C⋯H contacts add a 19.3% contribution to the Hirshfeld surface, with the tips at *d*_e_ + *d*_i_ ∼ 2.72 Å. H⋯O/O⋯H contacts make a 22.6% contribution to the Hirshfeld surface and are represented by a pair of sharp spikes in the region *d*_e_ + *d*_i_ ∼ 2.70 Å in the fingerprint plot. H⋯O/O⋯H inter­actions arise from inter­molecular O—H⋯O hydrogen bonding and C—H⋯O contacts. The contributions of the other contacts to the Hirshfeld surface are negligible, *i.e.* C⋯N/N⋯C of 3.0% and H⋯N/N⋯H of 5.8%.

## Synthesis and crystallization   

To a solution of 6-(4-hy­droxy-3-meth­oxy­phen­yl)-4-oxohex-5-enoic acid (0.25 g, 1 mmol) in 20 ml of ethanol, an equimolar amount of hydrazine hydrate was added. The mixture was maintained under reflux until thin-layer chromatography (TLC) indicated the end of the reaction. After cooling, the precipitate which formed was filtered off, washed with ethanol and recrystallized from ethanol. Slow evaporation at room temperature led to the formation of single crystals of the title com­pound.

## Refinement   

Crystal data, data collection and structure refinement details are summarized in Table 2 ▸. H atoms on C atoms were placed in idealized positions and refined as riding, with C—H = 0.93–0.97 Å and *U*_iso_(H) = 1.5*U*_eq_(C) for methyl H atoms and 1.2*U*_eq_(C) otherwise. The NH and OH hydrogens were located in a difference Fourier map and were constrained with N—H = 0.86 Å and *U*_iso_(H) = 1.2*U*_eq_(N), and O—H = 0.86 Å and *U*_iso_(H) = 1.5*U*_eq_(O), using a riding model.

## Supplementary Material

Crystal structure: contains datablock(s) I, global. DOI: 10.1107/S2056989019014130/wm5521sup1.cif

Structure factors: contains datablock(s) I. DOI: 10.1107/S2056989019014130/wm5521Isup3.hkl

CCDC references: 1959568, 1959568

Additional supporting information:  crystallographic information; 3D view; checkCIF report

## Figures and Tables

**Table 1:** Hydrogen-bond geometry (Å, °)

*D*—H⋯*A*	*D*—H	H⋯*A*	*D*⋯*A*	*D*—H⋯*A*
O2—H2⋯O1^i^	0.82	1.86	2.671 (2)	168
N1—H1⋯O1^ii^	0.86	2.02	2.875 (3)	170
C13—H13*A*⋯O2^iii^	0.96	2.51	3.465 (3)	172
C13—H13*C*⋯O2^iv^	0.96	2.57	3.489 (4)	159

Symmetry codes: (i) 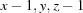; (ii) 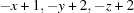; (iii) 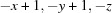; (iv) 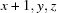.

**Table 2:** Experimental details

Crystal data	
Chemical formula	C_13_H_14_N_2_O_3_
*M*_r_	246.26
Crystal system, space group	Triclinic, *P* 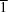
Temperature (K)	293
*a*, *b*, *c* (Å)	6.0828 (9), 9.4246 (13), 11.1724 (16)
α, β, γ (°)	75.838 (11), 83.099 (12), 84.059 (11)
*V* (Å^3^)	614.70 (16)
*Z*	2
Radiation type	Mo *K*α
μ (mm^−1^)	0.10
Crystal size (mm)	0.72 × 0.39 × 0.16

Data collection	
Diffractometer	Stoe IPDS 2
Absorption correction	Integration (*X-RED32*; Stoe & Cie, 2002 ▸)
*T*_min_, *T*_max_	0.944, 0.989
No. of measured, independent and observed [*I* > 2σ(*I*)] reflections	6563, 2426, 1506
*R*_int_	0.054
(sin θ/λ)_max_ (Å^−1^)	0.617

Refinement	
*R*[*F* ^2^ > 2σ(*F* ^2^)], *wR*(*F* ^2^), *S*	0.056, 0.147, 1.00
No. of reflections	2426
No. of parameters	165
H-atom treatment	H-atom parameters constrained
Δρ_max_, Δρ_min_ (e Å^−3^)	0.17, −0.17

Computer programs: *X-AREA* (Stoe & Cie, 2002 ▸), *X-RED* (Stoe & Cie, 2002 ▸), *SHELXT2017* (Sheldrick, 2015*a* ▸), *Mercury* (Macrae *et al.*, 2008 ▸), *PLATON* (Spek, 2009 ▸), *WinGX* (Farrugia, 2012 ▸), *SHELXL2018* (Sheldrick, 2015*b* ▸) and *publCIF* (Westrip, 2010 ▸).

## supplementary crystallographic information

### Crystal data

**Table:**

C_13_H_14_N_2_O_3_	*Z* = 2
*M**_r_* = 246.26	*F*(000) = 260
Triclinic, *P*1	*D*_x_ = 1.330 Mg m^−^^3^
*a* = 6.0828 (9) Å	Mo *K*α radiation, λ = 0.71073 Å
*b* = 9.4246 (13) Å	Cell parameters from 13077 reflections
*c* = 11.1724 (16) Å	θ = 2.2–30.7°
α = 75.838 (11)°	µ = 0.10 mm^−^^1^
β = 83.099 (12)°	*T* = 293 K
γ = 84.059 (11)°	Prism, yellow
*V* = 614.70 (16) Å^3^	0.72 × 0.39 × 0.16 mm

### Data collection

**Table:**

Stoe IPDS 2 diffractometer	1506 reflections with *I* > 2σ(*I*)
Detector resolution: 6.67 pixels mm^-1^	*R*_int_ = 0.054
rotation method scans	θ_max_ = 26.0°, θ_min_ = 2.2°
Absorption correction: integration (*X-RED32*; Stoe & Cie, 2002)	*h* = −7→7
*T*_min_ = 0.944, *T*_max_ = 0.989	*k* = −11→11
6563 measured reflections	*l* = −13→13
2426 independent reflections	

### Refinement

**Table:**

Refinement on *F*^2^	0 restraints
Least-squares matrix: full	Hydrogen site location: inferred from neighbouring sites
*R*[*F*^2^ > 2σ(*F*^2^)] = 0.056	H-atom parameters constrained
*wR*(*F*^2^) = 0.147	*w* = 1/[σ^2^(*F*_o_^2^) + (0.0747*P*)^2^] where *P* = (*F*_o_^2^ + 2*F*_c_^2^)/3
*S* = 1.00	(Δ/σ)_max_ < 0.001
2426 reflections	Δρ_max_ = 0.17 e Å^−^^3^
165 parameters	Δρ_min_ = −0.17 e Å^−^^3^

### Special details

**Table:**

Geometry. All esds (except the esd in the dihedral angle between two l.s. planes) are estimated using the full covariance matrix. The cell esds are taken into account individually in the estimation of esds in distances, angles and torsion angles; correlations between esds in cell parameters are only used when they are defined by crystal symmetry. An approximate (isotropic) treatment of cell esds is used for estimating esds involving l.s. planes.

### Fractional atomic coordinates and isotropic or equivalent isotropic displacement parameters (Å^2^)

**Table:**

	*x*	*y*	*z*	*U*_iso_*/*U*_eq_	
O1	0.7518 (3)	0.88682 (19)	0.99156 (15)	0.0601 (5)	
O3	0.4960 (3)	0.58008 (19)	0.11563 (16)	0.0614 (5)	
O2	0.1290 (3)	0.7317 (2)	0.05778 (16)	0.0636 (5)	
H2	0.016474	0.786724	0.044599	0.095*	
N1	0.4947 (3)	0.9323 (2)	0.85757 (17)	0.0525 (5)	
H1	0.429651	0.995567	0.896673	0.063*	
N2	0.3918 (3)	0.9139 (2)	0.75870 (17)	0.0519 (5)	
C9	0.3982 (4)	0.6576 (2)	0.1986 (2)	0.0497 (6)	
C5	0.3927 (4)	0.8291 (3)	0.5807 (2)	0.0506 (6)	
H5	0.252921	0.878478	0.572201	0.061*	
C1	0.6829 (4)	0.8631 (3)	0.8987 (2)	0.0492 (6)	
C4	0.5043 (4)	0.8412 (2)	0.6851 (2)	0.0472 (6)	
C8	0.4803 (4)	0.6645 (3)	0.3066 (2)	0.0521 (6)	
H8	0.616062	0.614251	0.326018	0.062*	
C11	0.0780 (4)	0.8118 (3)	0.2494 (2)	0.0538 (6)	
H11	−0.059111	0.860442	0.230853	0.065*	
C7	0.3664 (4)	0.7445 (2)	0.3875 (2)	0.0496 (6)	
C10	0.1954 (4)	0.7364 (2)	0.1681 (2)	0.0487 (6)	
C6	0.4707 (4)	0.7544 (3)	0.4962 (2)	0.0549 (6)	
H6	0.607897	0.702171	0.507391	0.066*	
C12	0.1607 (4)	0.8164 (3)	0.3580 (2)	0.0552 (6)	
H12	0.078822	0.867751	0.411814	0.066*	
C13	0.7007 (4)	0.4965 (3)	0.1419 (3)	0.0614 (7)	
H13A	0.745228	0.441469	0.079994	0.092*	
H13B	0.682204	0.430200	0.222071	0.092*	
H13C	0.812721	0.561367	0.141331	0.092*	
C2	0.7972 (5)	0.7565 (3)	0.8299 (3)	0.0694 (8)	
H2A	0.761992	0.658432	0.875007	0.083*	
H2B	0.956271	0.761159	0.827335	0.083*	
C3	0.7375 (4)	0.7807 (3)	0.7002 (2)	0.0673 (8)	
H3A	0.836608	0.847839	0.645178	0.081*	
H3B	0.760060	0.688125	0.675416	0.081*	

### Atomic displacement parameters (Å^2^)

**Table:**

	*U*^11^	*U*^22^	*U*^33^	*U*^12^	*U*^13^	*U*^23^
O1	0.0632 (11)	0.0804 (12)	0.0479 (10)	0.0136 (8)	−0.0292 (8)	−0.0331 (8)
O3	0.0642 (11)	0.0739 (11)	0.0579 (10)	0.0218 (8)	−0.0273 (9)	−0.0392 (9)
O2	0.0633 (12)	0.0859 (13)	0.0537 (10)	0.0146 (9)	−0.0326 (9)	−0.0349 (9)
N1	0.0536 (12)	0.0686 (13)	0.0448 (11)	0.0094 (9)	−0.0204 (9)	−0.0299 (10)
N2	0.0510 (12)	0.0670 (13)	0.0450 (11)	0.0068 (9)	−0.0213 (9)	−0.0237 (10)
C9	0.0569 (14)	0.0515 (13)	0.0479 (13)	0.0029 (11)	−0.0190 (11)	−0.0217 (11)
C5	0.0550 (14)	0.0602 (14)	0.0422 (12)	0.0029 (11)	−0.0196 (11)	−0.0185 (11)
C1	0.0539 (14)	0.0552 (14)	0.0427 (12)	0.0056 (11)	−0.0182 (11)	−0.0172 (11)
C4	0.0524 (14)	0.0522 (13)	0.0416 (12)	0.0007 (10)	−0.0152 (11)	−0.0168 (10)
C8	0.0533 (14)	0.0584 (14)	0.0511 (14)	0.0074 (11)	−0.0248 (12)	−0.0207 (11)
C11	0.0495 (13)	0.0675 (15)	0.0506 (14)	0.0090 (11)	−0.0211 (11)	−0.0237 (12)
C7	0.0579 (14)	0.0565 (14)	0.0412 (12)	0.0011 (11)	−0.0181 (11)	−0.0204 (11)
C10	0.0540 (14)	0.0560 (14)	0.0432 (13)	−0.0004 (11)	−0.0192 (11)	−0.0202 (11)
C6	0.0601 (15)	0.0640 (15)	0.0467 (13)	0.0033 (12)	−0.0229 (12)	−0.0197 (12)
C12	0.0558 (15)	0.0681 (15)	0.0490 (14)	0.0056 (12)	−0.0149 (12)	−0.0274 (12)
C13	0.0656 (16)	0.0634 (15)	0.0600 (16)	0.0144 (12)	−0.0203 (13)	−0.0248 (13)
C2	0.0734 (18)	0.0867 (19)	0.0599 (16)	0.0316 (14)	−0.0365 (14)	−0.0406 (14)
C3	0.0576 (16)	0.098 (2)	0.0593 (16)	0.0136 (14)	−0.0231 (13)	−0.0423 (15)

### Geometric parameters (Å, º)

**Table:**

O1—C1	1.241 (3)	C8—H8	0.9300
O3—C9	1.362 (3)	C11—C10	1.377 (3)
O3—C13	1.424 (3)	C11—C12	1.379 (3)
O2—C10	1.355 (2)	C11—H11	0.9300
O2—H2	0.8200	C7—C12	1.396 (3)
N1—C1	1.331 (3)	C7—C6	1.462 (3)
N1—N2	1.387 (2)	C6—H6	0.9300
N1—H1	0.8600	C12—H12	0.9300
N2—C4	1.288 (3)	C13—H13A	0.9600
C9—C8	1.378 (3)	C13—H13B	0.9600
C9—C10	1.406 (3)	C13—H13C	0.9600
C5—C6	1.327 (3)	C2—C3	1.493 (3)
C5—C4	1.451 (3)	C2—H2A	0.9700
C5—H5	0.9300	C2—H2B	0.9700
C1—C2	1.480 (3)	C3—H3A	0.9700
C4—C3	1.486 (3)	C3—H3B	0.9700
C8—C7	1.394 (3)		
			
C9—O3—C13	117.98 (17)	O2—C10—C11	124.3 (2)
C10—O2—H2	109.5	O2—C10—C9	116.1 (2)
C1—N1—N2	127.3 (2)	C11—C10—C9	119.54 (19)
C1—N1—H1	116.4	C5—C6—C7	127.7 (2)
N2—N1—H1	116.4	C5—C6—H6	116.1
C4—N2—N1	117.42 (18)	C7—C6—H6	116.1
O3—C9—C8	126.0 (2)	C11—C12—C7	120.5 (2)
O3—C9—C10	115.23 (18)	C11—C12—H12	119.8
C8—C9—C10	118.8 (2)	C7—C12—H12	119.8
C6—C5—C4	126.4 (2)	O3—C13—H13A	109.5
C6—C5—H5	116.8	O3—C13—H13B	109.5
C4—C5—H5	116.8	H13A—C13—H13B	109.5
O1—C1—N1	120.4 (2)	O3—C13—H13C	109.5
O1—C1—C2	123.3 (2)	H13A—C13—H13C	109.5
N1—C1—C2	116.36 (19)	H13B—C13—H13C	109.5
N2—C4—C5	115.5 (2)	C1—C2—C3	114.4 (2)
N2—C4—C3	122.97 (19)	C1—C2—H2A	108.7
C5—C4—C3	121.5 (2)	C3—C2—H2A	108.7
C9—C8—C7	122.0 (2)	C1—C2—H2B	108.7
C9—C8—H8	119.0	C3—C2—H2B	108.7
C7—C8—H8	119.0	H2A—C2—H2B	107.6
C10—C11—C12	121.0 (2)	C4—C3—C2	113.3 (2)
C10—C11—H11	119.5	C4—C3—H3A	108.9
C12—C11—H11	119.5	C2—C3—H3A	108.9
C8—C7—C12	118.02 (19)	C4—C3—H3B	108.9
C8—C7—C6	118.9 (2)	C2—C3—H3B	108.9
C12—C7—C6	123.1 (2)	H3A—C3—H3B	107.7
			
C1—N1—N2—C4	−12.5 (4)	O3—C9—C10—O2	−3.3 (3)
C13—O3—C9—C8	0.8 (3)	C8—C9—C10—O2	176.6 (2)
C13—O3—C9—C10	−179.3 (2)	O3—C9—C10—C11	176.6 (2)
N2—N1—C1—O1	−177.3 (2)	C8—C9—C10—C11	−3.5 (4)
N2—N1—C1—C2	1.2 (4)	C4—C5—C6—C7	−177.5 (2)
N1—N2—C4—C5	−177.97 (19)	C8—C7—C6—C5	177.4 (3)
N1—N2—C4—C3	−1.5 (3)	C12—C7—C6—C5	0.0 (4)
C6—C5—C4—N2	−176.7 (3)	C10—C11—C12—C7	0.2 (4)
C6—C5—C4—C3	6.8 (4)	C8—C7—C12—C11	−2.2 (4)
O3—C9—C8—C7	−178.7 (2)	C6—C7—C12—C11	175.2 (2)
C10—C9—C8—C7	1.4 (4)	O1—C1—C2—C3	−159.6 (3)
C9—C8—C7—C12	1.4 (4)	N1—C1—C2—C3	21.9 (4)
C9—C8—C7—C6	−176.1 (2)	N2—C4—C3—C2	23.8 (4)
C12—C11—C10—O2	−177.4 (2)	C5—C4—C3—C2	−160.0 (2)
C12—C11—C10—C9	2.7 (4)	C1—C2—C3—C4	−32.7 (4)

### Hydrogen-bond geometry (Å, º)

**Table:**

*D*—H···*A*	*D*—H	H···*A*	*D*···*A*	*D*—H···*A*
O2—H2···O1^i^	0.82	1.86	2.671 (2)	168
N1—H1···O1^ii^	0.86	2.02	2.875 (3)	170
C13—H13*A*···O2^iii^	0.96	2.51	3.465 (3)	172
C13—H13*C*···O2^iv^	0.96	2.57	3.489 (4)	159

Symmetry codes: (i) *x*−1, *y*, *z*−1; (ii) −*x*+1, −*y*+2, −*z*+2; (iii) −*x*+1, −*y*+1, −*z*; (iv) *x*+1, *y*, *z*.

## References

[bb1] Abourichaa, S., Benchat, N., Anaflous, A., Melhaoui, A., Ben-Hadda, T., Oussaid, B., El Bali, B. & Bolte, M. (2003). *Acta Cryst.* E**59**, o802–o803.

[bb2] Akhtar, W., Shaquiquzzaman, M., Akhter, M., Verma, G., Khan, M. F. & Alam, M. M. (2016). *Eur. J. Med. Chem.* **123**, 256–281. 10.1016/j.ejmech.2016.07.061 27484513

[bb3] Asif, M. (2013). *Mini-Rev. Org. Chem.* **10**, 113–122.

[bb4] Asif, M. (2014). *Rev. Med. Chem.* **14**, 1093–1103. 10.2174/1389557514666141127143133 25429662

[bb5] Barberot, C., Moniot, A., Allart-Simon, I., Malleret, L., Yegorova, T., Laronze-Cochard, M., Bentaher, A., Médebielle, M., Bouillon, J. P., Hénon, E., SAPI, J., Velard, F. & Gérard, S. (2018). *Eur. J. Med. Chem.* **146**, 139–146. 10.1016/j.ejmech.2018.01.035 29407945

[bb6] Boukharsa, Y., Meddah, B., Tiendrebeogo, R. Y., Ibrahimi, A., Taoufik, J., Cherrah, Y., Benomar, A., Faouzi, M. E. A. & Ansar, M. (2016). *Med. Chem. Res.* **25**, 494–500.

[bb7] Daoui, S., Cinar, E. B., El Kalai, F., Saddik, R., Karrouchi, K., Benchat, N., Baydere, C. & Dege, N. (2019*b*). *Acta Cryst.* E**75**, 1352–1356. 10.1107/S2056989019011551 PMC6727051 31523465

[bb8] Daoui, S., Faizi, M. S. H., Kalai, F. E., Saddik, R., Dege, N., Karrouchi, K. & Benchat, N. (2019*a*). *Acta Cryst.* E**75**, 1030–1034. 10.1107/S2056989019008557 PMC6659327 31392019

[bb9] Dubey, S. & Bhosle, P. A. (2015). *Med. Chem. Res.* **24**, 3579–3598.

[bb10] El Kalai, F., Baydere, C., Daoui, S., Saddik, R., Dege, N., Karrouchi, K. & Benchat, N. (2019). *Acta Cryst.* E**75**, 892–895. 10.1107/S2056989019007424 PMC6658951 31391989

[bb11] Farrugia, L. J. (2012). *J. Appl. Cryst.* **45**, 849–854.

[bb12] Gökçe, M., Utku, S. & Küpeli, E. (2009). *Eur. J. Med. Chem.* **44**, 3760–3764. 10.1016/j.ejmech.2009.04.048 19535179

[bb13] Groom, C. R., Bruno, I. J., Lightfoot, M. P. & Ward, S. C. (2016). *Acta Cryst.* B**72**, 171–179. 10.1107/S2052520616003954 PMC4822653 27048719

[bb14] Karrouchi, K., Ansar, M., Radi, S., Saadi, M. & El Ammari, L. (2015). *Acta Cryst.* E**71**, o890–o891. 10.1107/S2056989015020071 PMC4645091 26594583

[bb15] Livermore, D., Bethell, R. C., Cammack, N., Hancock, A. P., Hann, M. M., Green, D., Lamont, R. B., Noble, S. A., Orr, D. C. & Payne, J. J. (1993). *J. Med. Chem.* **36**, 3784–3794. 10.1021/jm00076a005 7504733

[bb16] Macrae, C. F., Bruno, I. J., Chisholm, J. A., Edgington, P. R., McCabe, P., Pidcock, E., Rodriguez-Monge, L., Taylor, R., van de Streek, J. & Wood, P. A. (2008). *J. Appl. Cryst.* **41**, 466–470.

[bb17] Partap, S., Akhtar, M. J., Yar, M. S., Hassan, M. Z. & Siddiqui, A. A. (2018). *Bioorg. Chem.* **77**, 74–83. 10.1016/j.bioorg.2018.01.001 29334622

[bb18] Sheldrick, G. M. (2015*a*). *Acta Cryst.* A**71**, 3–8.

[bb19] Sheldrick, G. M. (2015*b*). *Acta Cryst.* C**71**, 3–8.

[bb20] Siddiqui, A. A., Mishra, R., Shaharyar, M., Husain, A., Rashid, M. & Pal, P. (2011). *Bioorg. Med. Chem. Lett.* **21**, 1023–1026. 10.1016/j.bmcl.2010.12.028 21211966

[bb21] Sönmez, M., Berber, I. & Akbaş, E. (2006). *Eur. J. Med. Chem.* **41**, 101–105. 10.1016/j.ejmech.2005.10.003 16293349

[bb22] Spek, A. L. (2009). *Acta Cryst.* D**65**, 148–155. 10.1107/S090744490804362X PMC2631630 19171970

[bb23] Stoe & Cie (2002). *X-AREA* and *X-RED32*. Stoe & Cie GmbH, Darmstadt, Germany.

[bb24] Tao, M., Aimone, L. D., Gruner, J. A., Mathiasen, J. R., Huang, Z., Lyons, J., Raddatz, R. & Hudkins, R. L. (2012). *Bioorg. Med. Chem. Lett.* **22**, 1073–1077. 10.1016/j.bmcl.2011.11.118 22197136

[bb25] Turner, M. J., McKinnon, J. J., Wolff, S. K., Grimwood, D. J., Spackman, P. R., Jayatilaka, D. & Spackman, M. A. (2017). *CrystalExplorer17*. University of Western Australia. http://hirshfeldsurface.net.

[bb26] Wang, T., Dong, Y., Wang, L.-C., Xiang, B.-R., Chen, Z. & Qu, L.-B. (2008). *Arzneimittelforschung*, **58**, 569–573. 10.1055/s-0031-1296558 19137907

[bb27] Westrip, S. P. (2010). *J. Appl. Cryst.* **43**, 920–925.

[bb28] Zhang, C.-T., Wu, J.-H., Zhou, L.-N., Wang, Y.-L. & Wang, J.-K. (2006). *Acta Cryst.* E**62**, o2999–o3000.

[bb29] Zhou, G., Ting, P. C., Aslanian, R., Cao, J., Kim, D. W., Kuang, R., Lee, J. F., Schwerdt, J., Wu, H., Jason Herr, R., Zych, A. J., Yang, J., Lam, S., Wainhaus, S., Black, T. A., McNicholas, P. M., Xu, Y. & Walker, S. S. (2011). *Bioorg. Med. Chem. Lett.* **21**, 2890–2893. 10.1016/j.bmcl.2011.03.083 21489787

